# Neuropsychiatric involvement in systemic lupus erythematosus contributes to organ damage beyond the nervous system: a post-hoc analysis of 5 phase III randomized clinical trials

**DOI:** 10.1007/s00296-024-05667-5

**Published:** 2024-08-08

**Authors:** Dionysis Nikolopoulos, Nursen Cetrez, Julius Lindblom, Ioannis Parodis

**Affiliations:** 1 Division of Rheumatology, Department of Medicine Solna, Karolinska Institutet, Stockholm, Sweden; 2 Department of Gastroenterology, Dermatology and Rheumatology, Karolinska University Hospital, 171 76 Stockholm, Sweden; 3 Department of Rheumatology, Faculty of Medicine and Health, Örebro University, Örebro, Sweden

**Keywords:** Systemic lupus erythematosus, Neuropsychiatric lupus, SLICC/ACR damage index, Outcomes, Nervous system

## Abstract

**Objective:**

To investigate the association between neuropsychiatric systemic lupus erythematosus (NPSLE) and SLICC/ACR damage index (SDI) items, especially non-neuropsychiatric items.

**Methods:**

Baseline data from five phase III trials (BLISS-52, BLISS-76, BLISS-SC, BLISS-NEA, EMBRACE) were analysed. NPSLE involvement was defined as NP BILAG A/B/C/D (n = 272); NP BILAG E denoted non-neuropsychiatric SLE (n = 3273). We employed multivariable logistic regression analysis adjusting for age, sex, disease duration, and ethnicity.

**Results:**

The median (IQR) and mean ± SD SDI scores were 0 (0–1) and 0.62 ± 1.09. Compared with the non-neuropsychiatric SLE group, NPSLE patients were more likely to develop damage (adjusted (a)OR = 2.86; 95% CI = 2.28–3.59). This held true also after suppression of the NP SDI items (aOR = 1.70; 95% CI = 1.36–2.12). Beyond the neuropsychiatric domain, NPSLE was associated with damage in the cardiovascular (aOR = 2.63; 95% CI = 1.75–3.95), musculoskeletal (aOR = 1.90; 95% CI = 1.43–2.52), and skin (aOR = 1.54; 95% CI = 1.06–2.22) SDI domains. Dissecting domains into items, NPSLE was associated with coronary artery disease (aOR = 3.08; 95% CI = 1.44–6.58), myocardial infraction (aOR = 3.11; 95% CI = 1.54–6.27), muscle atrophy (aOR = 3.34; 2.16–5.16), scarring alopecia (aOR = 1.79; 95% CI = 1.19–2.70), bowel infarction (aOR = 1.98; 95% CI = 1.20–3.26), retinopathy (aOR = 2.23; 95% CI = 1.15–4.32), and premature gonadal failure (aOR = 2.10; 95% CI = 1.11–3.90).

**Conclusion:**

The intricate association between NPSLE and damage accrual extends beyond the nervous system to also comprise the musculoskeletal, skin, and cardiovascular organ systems.

**Supplementary Information:**

The online version contains supplementary material available at 10.1007/s00296-024-05667-5.

## Introduction

Neuropsychiatric (NP) manifestations affecting either the central or peripheral nervous system occur in approximately 20% of patients with systemic lupus erythematosus (SLE) and are collectively referred to as neuropsychiatric SLE (NPSLE) [1]. Although NPSLE is not considered a rare condition within SLE, it remains one of the less understood aspects of the disease, particularly concerning diagnosis, treatment, and natural history [2]. This is largely due to the significant clinical heterogeneity, which hampers the generation of large homogeneous cohorts for studying this disease phenotype.

NPSLE is associated with adverse disease-related outcomes including poorer health-related quality of life (HRQoL) [3, 4], higher mortality [5], and increased accrual of irreversible organ damage compared with SLE patients without NP involvement [6]. Specifically, patients with NPSLE exhibit an estimated mortality that is approximately three times higher (16%) than in SLE patients without NP involvement (6%) within the first 10 years following diagnosis [5]. Furthermore, NPSLE patients accrue damage more frequently (76%) than patients without NP involvement (34%) over the first decade of diagnosis [7]. Importantly, NP involvement in SLE represents an independent risk factor for subsequent organ damage accrual [8]. However, our knowledge regarding the impact of NPSLE on damage accrual derives from few studies and limited sample sizes.

Recently, we used baseline data from four randomised clinical trials (RCTs) to investigate the impact of neuropsychiatric involvement on SLE patients’ HRQoL. We showed that patients with NPSLE –irrespective of NP activity– experience a more impaired HRQoL and are more fatigued compared with SLE patients without NP involvement [4]. In the present study, we aimed to determine the impact of NP involvement on organ damage in a large population of patients with SLE deriving from five phase III clinical trials. Apart from total organ damage, we also compared the frequency of organ damage across organs or organ systems between NPSLE patients and SLE patients with no NP involvement.

## Methods

### Study design and population

We performed a post-hoc analysis utilising baseline data from five double-blinded, placebo-controlled phase III trials of belimumab: BLISS-52 (NCT00424476) [9], BLISS-76 (NCT00410384) [10], BLISS-SC (NCT01484496) [11], BLISS-NEA (NCT01345253) [12], and EMBRACE (NCT01632241) [13]. These trials enrolled 865, 819, 839, 677, and 448 patients with SLE, respectively, yielding 3645 participants. All participants were aged 18 years or older and met the classification criteria for SLE according to the revised 1997 American College of Rheumatology (ACR) criteria [14].

In each trial, all patients were seropositive, defined as having an antinuclear antibody (ANA) titre of 1:80 or higher and/or an anti-double-stranded DNA antibody (anti-dsDNA) level of 30 IU/mL or higher. Moreover, all participants exhibited active SLE, as indicated by a Safety of Estrogens in Lupus National Assessment—Systemic Lupus Erythematosus Disease Activity Index (SELENA-SLEDAI) score of 6 or higher in BLISS-52 and BLISS-76, and a SELENA-SLEDAI score of 8 or higher in BLISS-SC, BLISS-NEA, and EMBRACE.

### Definition of neuropsychiatric SLE

For the current investigation, the SLE phenotype was classified as either NPSLE or non-neuropsychiatric SLE at the baseline (week 0) evaluation. The presence of neuropsychiatric involvement was determined based on the NP domain of the classic British Isles Lupus Assessment Group (BILAG) index and/or NP descriptors of the SLE Disease Activity Index 2000 (SLEDAI-2 K). NP involvement was defined as an NP BILAG score of A, B, C, or D and/or at least one NP descriptor scored in SLEDAI-2 K, resulting in 372 patients. Among patients with NPSLE, 298 individuals (80.1%) had NP BILAG scores C or D, with no neuropsychiatric descriptor scored in SLEDAI-2 K, indicating inactive NPSLE at the baseline evaluation. The remaining patients with NPSLE (N = 74; accounting for 19.9% of the NPSLE group) were classified based on either neuropsychiatric BILAG scores of A or B, or NP descriptors scored in SLEDAI-2 K, signifying active NPSLE at the baseline evaluation. The non-neuropsychiatric SLE cohort comprised patients with an NP BILAG score of E (N = 3273).

### Evaluation of irreversible organ damage in SLE

Irreversible organ damage in SLE was assessed using the Systemic Lupus International Collaborating Clinics (SLICC)/ACR Damage Index (SDI) [15]. SDI scores were provided separately for all SDI categories as well as individual SDI items within each category, including neuropsychiatric (cognitive impairment, seizure requiring therapy for at least 6 months, cerebrovascular accident, cranial or peripheral neuropathy, and transverse myelitis), renal (estimated or measured glomerular filtration rate (GFR) < 50 mL/min, proteinuria > 3.5 g per 24 h, and end-stage kidney disease), pulmonary (pulmonary hypertension, pulmonary fibrosis, shrinking lung, pleural fibrosis, and pulmonary infarction), musculoskeletal (muscle atrophy or weakness, deforming or erosive arthritis, osteoporosis with fracture or vertebral collapse, avascular necrosis, and osteomyelitis), ocular (cataract, and retinal change or optic atrophy), cardiovascular (angina or coronary artery bypass, myocardial infraction, cardiomyopathy, valvular disease, and pericarditis for 6 months or pericardiectomy), peripheral vascular (claudication for 6 months, minor tissue loss in pulp space, significant tissue loss ever, venous thrombosis, mesenteric insufficiency, and infarction or resection of bowel), skin (scarring chronic alopecia, extensive scarring of panniculum other than scalp and pulp space and skin ulceration for at least 6 months), gastrointestinal (chronic peritonitis, and stricture or upper gastrointestinal tract surgery ever), premature gonadal failure, diabetes, and malignancy.

### Statistical analysis

Descriptive statistics are presented in the form of numbers and percentages or means ± standard deviation (SD). In cases where distributions were non-normal, medians and interquartile range are provided. The Mann–Whitney *U* test was used for comparing unrelated continuous data. For assessing associations between unrelated binomial variables, either the Pearson’s chi-squared test or the Fisher’s exact test was employed, as appropriate. Logistic regression analysis investigated associations between NP involvement and damage in individual SDI items or SDI categories, with univariable analysis being employed when events were fewer than 30, and multivariable analysis accounting for age, sex, disease duration, and ancestry in all other cases. All analyses were repeated after exclusion of patient with the headache descriptor scored in BILAG or SLEDAI-2 K from analysis. Statistical significance was determined by p-values < 0.05. All analyses were performed using the R software version 4.1.0, developed by the R Foundation for Statistical Computing in Vienna, Austria.

### Ethics

GlaxoSmithKline, based in Uxbridge, UK, provided access to data from the clinical trials through the Clinical Study Data Request consortium. The study upheld ethical principles as outlined in the Declaration of Helsinki. Prior to enrolment, written informed consent was obtained from all participants. Approval for the study protocols of BLISS-52, BLISS-76, BLISS-SC, BLISS-NEA, and EMBRACE was granted by regional ethics review boards for all participating centres. Moreover, the protocol for the current study received approval from the Swedish Ethical Review Authority under reference number 2019–05498.

## Results

### Demographics and clinical characteristic

The demographics of NPSLE and non-neuropsychiatric SLE groups are presented in Table 1. Most participants were women (94.3%). The mean (± SD) age of study participants at baseline was 37.0 years (± 11.6). The NPSLE and non-neuropsychiatric SLE groups did not differ with regard to sex or extra-neuropsychiatric disease activity based on SLEDAI-2 K, but patients with NPSLE were older than non-neuropsychiatric SLE individuals (40.2 ± 11.0 versus 36.6 ± 11.6 years; p < 0.001). Moreover, NPSLE patients were more likely to be White/Caucasians (54% versus 33.6%; p < 0.001) and less likely to be Asians (13.4% versus 35.6%; p < 0.001) compared with SLE patients without NP involvement.

**Table 1:** Demographics and clinical features of SLE patients in the pooled study population

	All patients (N = 3545)	Non-NP SLE (N = 3273)	NPSLE (N = 372)	*p* value
Demographics				
Age; mean (S.D.)	37.0 (11.6)	36.6 (11.6)	40.6 (11.0)	** < 0.001**
Female sex; n (%)	3437 (94.3)	3086 (94.3)	351 (94.4)	1.000
Ethnicity; n (%)				
Asian	1214 (33.3)	1164 (35.6)	50 (13.4)	** < 0.001**
Black/African American	680 (18.8)	604 (18.5)	76 (20.4)	0.392
Indigenous American^a^	451 (12.4)	406 (12.4)	45 (12.1)	0.930
White/Caucasian	1300 (35.7)	1099 (33.6)	201 (54.0)	** < 0.001**
**Clinical features**				
SLE disease duration; mean (S.D.)	6.44 (6.3)	6.31 (6.2)	7.61 (6.9)	**0.002**
SDI score; mean (S.D.)	0.62 (1.1)	0.54 (1.0)	1.32 (1.5)	** < 0.001**
Extra-NP SDI score; mean (S.D.)	0.52 (0.9)	0.48 (0.9)	0.86 (1.2)	** < 0.001**
SLEDAI-2 K; mean (S.D.)	10.32 (3.7)	10.22 (3.6)	11.19 (4.5)	** < 0.001**
Extra-NP SLEDAI-2 K; mean (S.D.)	10.18 (3.6)	10.22 (3.6)	9.84 (4.1)	0.052
Clinical SLEDAI-2 K; mean (S.D.)	7.75 (3.5)	7.61 (3.4)	9.01 (4.3)	** < 0.001**
Non-NP Clinical SLEDAI-2 K; mean (S.D.)	7.61 (3.5)	7.61 (3.4)	7.66 (3.7)	0.788

Data are presented as numbers (percentage) or means (standard deviation). Statistically significant *p* values are in bold. ^a^Alaska Native or American Indian from North, South or Central America. *NA* not applicable; *NP* neuropsychiatric; *NPSLE* neuropsychiatric systemic lupus erythematosus; *S.D*. standard deviation; *SDI* Systemic Lupus International Collaborating Clinics (SLICC)/American College of Rheumatology (ACR) Damage Index; *SLE* systemic lupus erythematosus; *SLEDAI-2 K* Systemic Lupus Erythematosus Disease Activity Index 2000

The neuropsychiatric manifestations of SLE patients are presented in Table 2. Although severe active CNS involvement at the time of screening was an exclusion criterion by the trial protocols, we identified 74 patients with active NPSLE at baseline (week 0), among whom 17 patients exhibited severe NPSLE according to the trial protocol definitions (seizures, psychosis, organic brain syndrome, cerebrovascular accident, cerebritis, or CNS vasculitis; uniform definition across all trials); this may depend on changes in status between the screening phase and the baseline evaluation. Lupus headache, cranial and peripheral neuropathies, and depression were the most common NP manifestations.

**Table 2:** Neuropsychiatric events in the entire NPSLE population

	All NPSLE patients (N = 372)	Inactive NPSLE (N = 298)	Active NPSLE (N = 74)	*p* value
Neuropsychiatric events				
Transverse myelitis; n (%)	1 (0.3)	1 (0.3)	0 (0.0)	1.000
Aseptic meningitis; n (%)	0 (0.0)	0 (0.0)	0 (0.0)	NA
Movement disorder; n (%)	11 (2.9)	4 (1.1)	7 (9.5)	**0.035**
Mononeuritis multiplex; n (%)	8 (2.3)	8 (2.6)	0 (0.0)	0.463
Psychosis; n (%)	4 (1.1)	0 (0.0)	4 (5.6)	**0.001**
Seizures; n (%)	1 (0.3)	0 (0.0)	1 (1.4)	NA
Depression, n (%)	52 (14.9)	39 (14.0)	13 (13.8)	0.466
Stroke; n (%)	2 (0.6)	1 (0.4)	1 (1.4)	0.868
Cranial neuropathy; n (%)	7 (1.9)	0 (0.0)	7 (9.5)	** < 0.001**
Optic neuritis; n (%)	11 (3.0)	0 (0.0)	11 (14.9)	** < 0.001**
Headache; n (%)	220 (59.1)	175 (58.7)	45 (60.8)	0.934
Acute confusional state; n (%)	17 (4.7)	6 (2.0)	11 (14.9)	** < 0.001**
Polyneuropathy; n (%)	77 (20.7)	56 (18.8)	21 (28.4)	0.053

Data are presented as numbers (percentage). Statistically significant *p* values are in bold. *BILAG* British Isles Lupus Assessment Group; *NA* not applicable; *NPSLE* neuropsychiatric systemic lupus erythematosus; *SLEDAI* Systemic Lupus Erythematosus Disease Activity Index

### Neuropsychiatric involvement in SLE is associated with organ damage

Patients with NPSLE had greater total SDI scores (mean ± SD) compared with the non-neuropsychiatric SLE group (1.32 ± 1.50 vs. 0.54 ± 1.00; p < 0.001; Table 1), even after the exclusion of neuropsychiatric items from the total SDI score (0.86 ± 1.20 vs. 0.48 ± 0.90; p < 0.001; Table 1). Moreover, NPSLE patients were more likely to have developed irreversible damage compared with non-neuropsychiatric SLE patients (adjusted (a)OR: 2.86; 95% CI: 2.28–3.59), the latter holding true also after suppression of the NP SDI items (aOR: 1.70; 95% CI: 1.36–2.12).

### NPSLE is associated with neuropsychiatric and cardiovascular damage

**Fig. 1:**
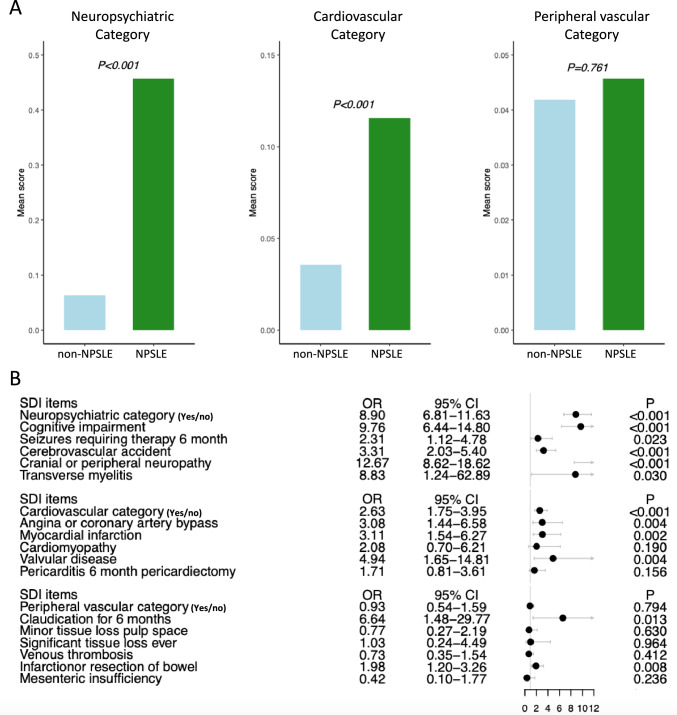
Associations between NPSLE and neuropsychiatric, cardiovascular, and vascular damage. (**A**) Bar plots illustrating the mean (± SD) SDI score in the neuropsychiatric, cardiovascular, and vascular SDI domains between NPSLE (green) and non-neuropsychiatric SLE (blue) patients. The Mann–Whitney *U* test was applied to compare the two groups. (**B**) Forest plot showing the odds ratios (ORs) and 95% confidence intervals (CIs) deriving from logistic regression analysis adjusting for age, sex, disease duration, and ethnicity. A higher OR indicates greater risk of established organ damage. *CI* confidence interval; *NPSLE *neuropsychiatric systemic lupus erythematosus; *OR* odds ratio; *SD* standard deviation; *SDI* Systemic Lupus International Collaborating Clinics (SLICC)/American College of Rheumatology (ACR) Damage Index; *SLE* Systemic Lupus Erythematosus

Patients with NPSLE had greater NP SDI scores (0.46 ± 0.68 versus. 0.06 ± 0.29; p < 0.001; Fig. 1A) and were more likely to have developed NP damage (aOR: 8.90; 95% CI: 6.81–11.63) compared with the non-neuropsychiatric SLE group (Fig. 1B). As expected, NP involvement was associated with more frequently scored SDI items within the neuropsychiatric domain (p < 0.05 for all SDI items; Fig. 1B). More importantly, neuropsychiatric involvement was associated with both greater SDI scores (0.12 ± 0.39 versus 0.04 ± 0.22; p < 0.001; Fig. 1A) and accrued damage in the cardiovascular domain (aOR: 2.63; 95% CI: 1.75–3.95; Fig. 1B), but not the peripheral vascular domain (aOR: 0.93; 95% CI: 0.54–1.59; Fig. 1B). Dissecting the SDI domains into specific items, neuropsychiatric involvement was associated with coronary artery disease (aOR: 3.08; 95% CI: 1.44–6.58), myocardial infraction (aOR: 3.11; 95% CI: 1.54–6.27), and valvular disease (OR: 4.94; 95% CI: 1.65–14.81; Fig. 1B). Moreover, NPSLE patients were more likely to develop claudication (OR: 6.64; 95% CI: 1.48–29.77; Fig. 1B) and bowel infraction (aOR: 1.98; 95% CI: 1.20–3.26; Fig. 1B), both items within the peripheral vascular SDI domain. It is worth noting that similar results were documented after exclusion of cases of headache from the NPSLE group (Supplementary Figure S1).

### Neuropsychiatric involvement in SLE is associated with skin and musculoskeletal damage

**Fig. 2:**
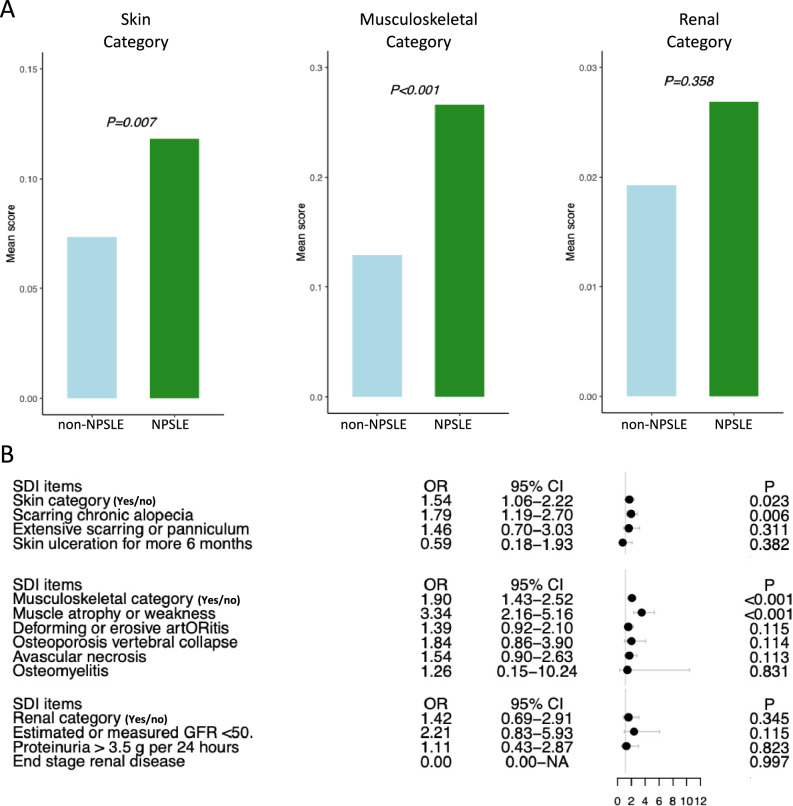
Associations between NPSLE and skin, musculoskeletal, and renal damage. (**A**) Bar plots illustrating the mean (± SD) SDI score in the skin, musculoskeletal, and renal SDI domains between NPSLE (green) and non-neuropsychiatric SLE (blue) patients. The Mann–Whitney *U* test was applied to compare the two groups. (**B**) Forest plot showing the odds ratios (ORs) and 95% confidence intervals (CIs) deriving from logistic regression analysis adjusting for age, sex, disease duration, and ethnicity. A higher OR indicates greater risk of established organ damage. *CI* confidence interval; *NPSLE* neuropsychiatric systemic lupus erythematosus; *OR* odds ratio; *SD* standard deviation; *SDI* Systemic Lupus International Collaborating Clinics (SLICC)/American College of Rheumatology (ACR) Damage Index; *SLE *Systemic Lupus Erythematosus

NPSLE was associated with increased SDI scores in the skin (0.12 ± 0.36 versus 0.07 ± 0.30; p = 0.007; Fig. 2A) and musculoskeletal (0.27 ± 0.59 versus 0.13 ± 0.41; p < 0.001; Fig. 2A) domains but not the renal SDI domain (0.03 ± 0.18 vs. 0.02 ± 0.15; p = 0.358; Fig. 2A). Moreover, neuropsychiatric involvement was associated with more frequently scored SDI items within the skin (aOR: 1.54; 95% CI: 1.06–2.22) and musculoskeletal (aOR: 1.90; 95% CI: 1.43–2.52) domains, but no such association was observed in the renal domain (aOR: 1.42; 95% CI: 0.69–2.91; Fig. 2B). The significant associations were driven by muscle atrophy or weakness within the musculoskeletal domain (aOR: 3.34; 95% CI: 2.16–5.16) and by scarring chronic alopecia within the skin domain (aOR: 1.78; 95% CI: 1.19–2.70; Fig. 2B). These results remained significant after exclusion of cases of headache from the NPSLE group (Supplementary Figure S2).

### Neuropsychiatric involvement in SLE is associated with ocular damage

**Fig. 3:**
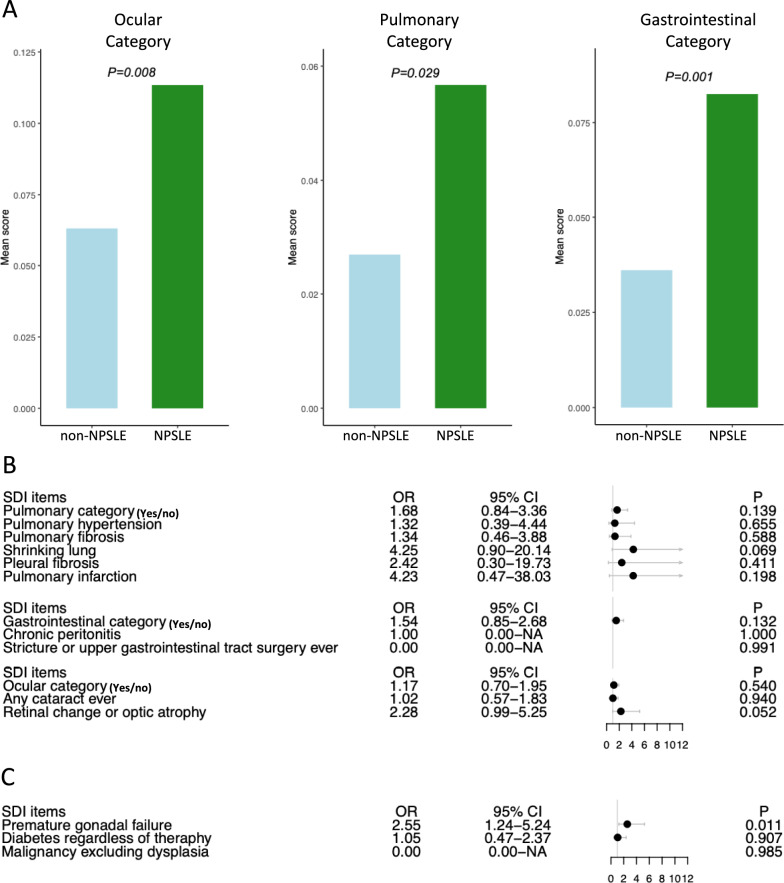
Associations between NPSLE and ocular, pulmonary, gastrointestinal damage, premature gonadal failure, diabetes, and malignancies. (**A**) Bar plots illustrating the mean (± SD) SDI score in the ocular, pulmonary, and gastrointestinal SDI domains between NPSLE (green) and non-neuropsychiatric SLE (blue) patients. The Mann–Whitney *U* test was applied to compare the two groups. (**B, C**) Forest plot showing the odds ratios (ORs) and 95% confidence intervals (CIs) deriving from logistic regression analysis adjusting for age, sex, disease duration, and ethnicity. A higher OR indicates greater risk of established organ damage. *CI* confidence interval; *NPSLE* neuropsychiatric systemic lupus erythematosus; *OR *odds ratio; *SD* standard deviation; *SDI* Systemic Lupus International Collaborating Clinics (SLICC)/American College of Rheumatology (ACR) Damage Index; *SLE* Systemic Lupus Erythematosus

Moreover, we investigated associations between NPSLE and damage within less commonly affected organ systems, including ocular, pulmonary, and gastrointestinal damage. We observed higher SDI scores in the ocular domain in NPSLE versus non-neuropsychiatric SLE patients (0.11 ± 0.34 versus 0.06 ± 0.25; p = 0.002; Fig. 3A), while no statistically significant difference was seen between the two groups within the gastrointestinal SDI domain (0.05 ± 0.22 versus 0.03 ± 0.18; p = 0.063; Fig. 3A). Moreover, neuropsychiatric involvement was associated with damage in the ocular (aOR: 1.49; 95% CI: 1.02–2.18) but not the gastrointestinal SDI domain (aOR: 1.36; 95% CI: 0.85–2.17; Fig. 3B). Although patients with NPSLE had higher SDI scores within the pulmonary domain (0.06 ± 0.24 versus 0.04 ± 0.19; p = 0.016; Fig. 3A), this finding did not hold true in regression analysis (aOR: 1.61; 95% CI: 0.92–2.80; Fig. 3B). The association between NPSLE and ocular damage was mainly driven by retinal change or optic atrophy (aOR: 2.23; 95% CI: 1.15–4.32; Fig. 3B). After exclusion of cases of headache from the NPSLE group, we did not observe statistically significant associations between NPSLE and ocular, pulmonary or gastrointestinal damage in logistic regression analysis (Supplementary Figure S3).

### Neuropsychiatric involvement in SLE is associated with premature gonadal failure

For the one-item domains premature gonadal failure, diabetes, and malignancy, we only performed logistic regression analysis. In this analysis, NPSLE was associated with premature gonadal failure (aOR: 2.10; 95% CI: 1.13–3.90) but not diabetes or malignancies (Fig. 3C). None of NPSLE patients had been diagnosed with any type of malignancy prior to enrolment. These results remained similar after exclusion of cases of headache from the NPSLE group (Supplementary Figure S3).

## Discussion

In the present study, we used baseline data from five RCTs to investigate the association between NP involvement in SLE and irreversible organ damage assessed by SDI. We demonstrated that patients with NPSLE accrue damage to a greater extent than SLE patients without NP involvement, a pattern that persisted even after excluding the NPSLE-specific SDI items from the total SDI score. Of note, we showed that patients with NPSLE had a propensity to accumulate organ damage beyond SDI items related to NP manifestations, i.e., cardiovascular, skin, and musculoskeletal damage.

The presence of antiphospholipid (aPL) antibodies is strongly associated with specific neuropsychiatric events in SLE [16]; thus, aPL antibodies are present to a greater extent in NPSLE patients compared with SLE patients without NP involvement [17]. Given that aPL antibodies are independent risk factors for both venous and arterial thrombosis [18], it is anticipated that NPSLE patients tend to accrue damage attributed to thrombosis-related items to a greater extent than SLE patients without NP involvement. Indeed, we observed that NPSLE patients accrued cardiovascular damage more frequently than SLE patients without NP involvement, mainly attributed to myocardial infraction, angina, and claudication. Moreover, we detected a significant association between NPSLE and valvular disease, which also constitutes a manifestation linked to aPL antibodies and was recently included in the 2023 classification criteria for antiphospholipid syndrome [19]. This finding provides additional evidence supporting the heightened aPL antibody-mediated signalling within the NPSLE group. However, the full aPL antibody profile (anti-cardiolipidin, anti-β_2_-glycoprotein I, lupus anticoagulant) was not available for all participants and, therefore, we could not determine whether these results were exclusively driven by aPL antibodies. To this end, we reason that a higher frequency of aPL antibodies in the NPSLE population may account –at least in part– for the increased frequency of cardiovascular and vascular damage in these patients.

Beyond NP and vascular damage, NP involvement in our study was also associated with damage in the skin and musculoskeletal domains. The former association was driven by scarring alopecia and may be considered an expected finding given the known higher frequency of skin manifestations in patients with NPSLE [20]. The latter observation was driven by muscle atrophy/weakness. Damage in this item may be attributed to the use of glucocorticoids [21], which are often administered at high doses to manage NPSLE [22]. This is further supported by the increased frequency of premature gonadal failure in NPSLE, which is typically a side-effect of cyclophosphamide [23], suggesting that the NPSLE group had been treated with potent immunosuppression and thus to manage their severe disease phenotype. However, we were unable to capture differences concerning the prevalence of diabetes between the two groups, which also is known to occur more frequently in patients consuming high doses of glucocorticoids over a longer term [24]. Unfortunately, we did not have historical data on treatments prior to baseline.

Albeit seldom affected in SLE, NPSLE in our study was associated with damage accumulation within the ocular and pulmonary domains, particularly due to damage from retinal changes or optic atrophy and shrinking lung, respectively. Although shrinking lung syndrome is a poorly understood manifestation of SLE due to its rarity, it is believed that it occurs in the context of phrenic neuropathy [25]; hence, affliction of the peripheral nervous system might be a contributing factor. Along similar lines, ocular damage could either arise as a result of optic neuritis or retinal vasculitis as a primary NP manifestation of SLE [26, 27], or as a result of chronic exposure to antimalarial agents such as hydroxychloroquine [28].

Being a retrospective investigation of clinical trial data, our study had limitations regarding its design. Firstly, the belimumab trial protocols excluded patients with active CNS vasculitis and severe NPSLE, such as seizures. Although this referred to the screening phase of the trials and not to historical NPSLE activity, the findings might be different for severe active forms of NPSLE. Secondly, the prevalence of headaches was high [29], accounting for 1% of the active NPSLE cases and 7% of all NPSLE cases. To mitigate this, we performed a subgroup analysis after exclusion of patients with headache at baseline, as per BILAG or SLEDAI-2 K; reassuringly, this analysis yielded similar associations to those seen in the entire NPSLE population including headache. Lastly, data on certain potential confounding factors such as socioeconomic status or information regarding complete aPL antibody profiles were unavailable. However, a significant strength of our study lied in the large study population and datasets, which allowed us to investigate associations with individual SDI items and SDI domains, yielding a comprehensive characterisation of irreversible organ damage in this SLE population and enhancing the novelty and overall integrity of the study.

In conclusion, NP affliction in SLE contributes to accrual of organ damage that extends beyond the realm of the nervous system, also including the musculoskeletal, skin, and cardiovascular organ domains. Further research is required to determine the precise relationship between NPSLE and the various components of SDI and shed light on how specific organs or tissues are impacted by NP manifestations. Elucidating this association would enhance our understanding of the disease and possibly guide the development of targeted, less toxic therapies for treating SLE with or without neuropsychiatric manifestations, thus minimising overall organ damage.

### Supplementary Information

Below is the link to the electronic supplementary material.

Supplementary file1 (PDF 209 KB)

Supplementary file2 (PDF 196 KB)

Supplementary file3 (PPTX 246 KB)

## Data Availability

The datasets used and analysed during the present study can be made available through the Clinical Study Data Request consortium.

## Declarations

### Clinical trial registration

The trials of belimumab, including BLISS-52, BLISS-76, BLISS-SC, BLISS-NEA, and EMBRACE, were registered on ClinicalTrials.gov with the following identifiers: NCT00424476, NCT00410384, NCT01484496, NCT01345253, and NCT01632241, respectively.
