# Supplementary figures and images for: Neuropsychiatric involvement in systemic lupus erythematosus contributes to organ damage beyond the nervous system: a post-hoc analysis of 5 phase III randomized clinical trials
Source: Rheumatol Int. 2024 Aug 8;44(9):1679–89. doi: 10.1007/s00296-024-05667-5 (PMC11343782; doi:10.1007/s00296-024-05667-5)

A

Neuropsychiatric  
Category

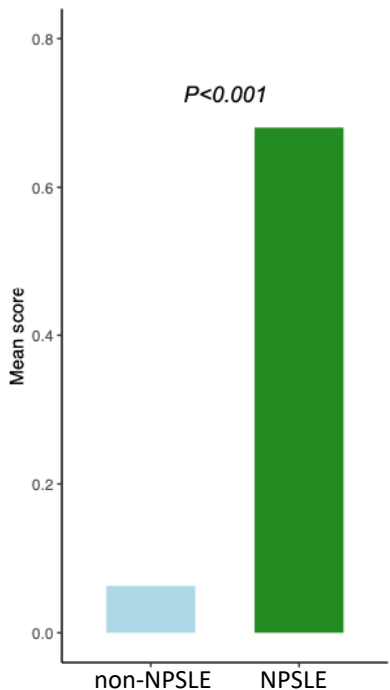

Cardiovascular  
Category

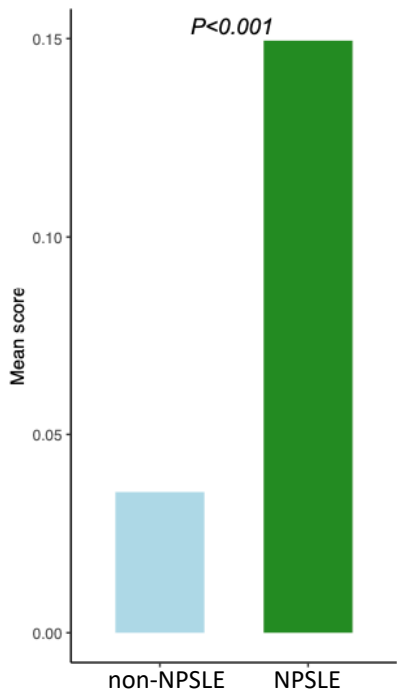

Peripheral vascular  
Category

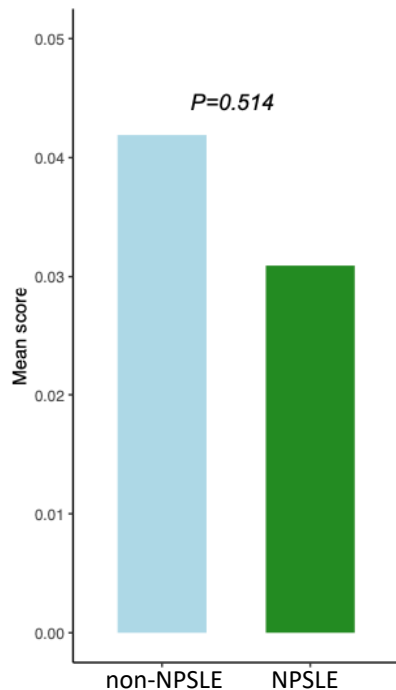

B

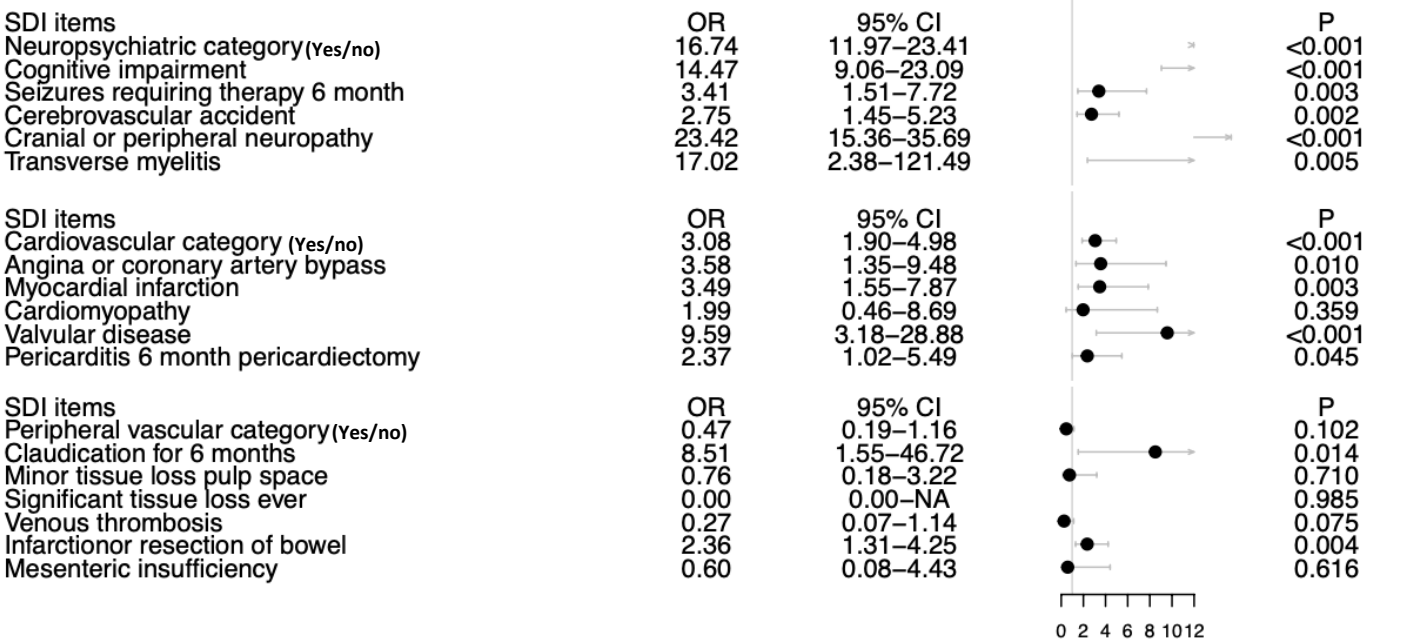

Supplement: Supplementary file 1 — Supplementary file1 (PDF 209 KB) [file 296_2024_5667_MOESM1_ESM.pdf]

A

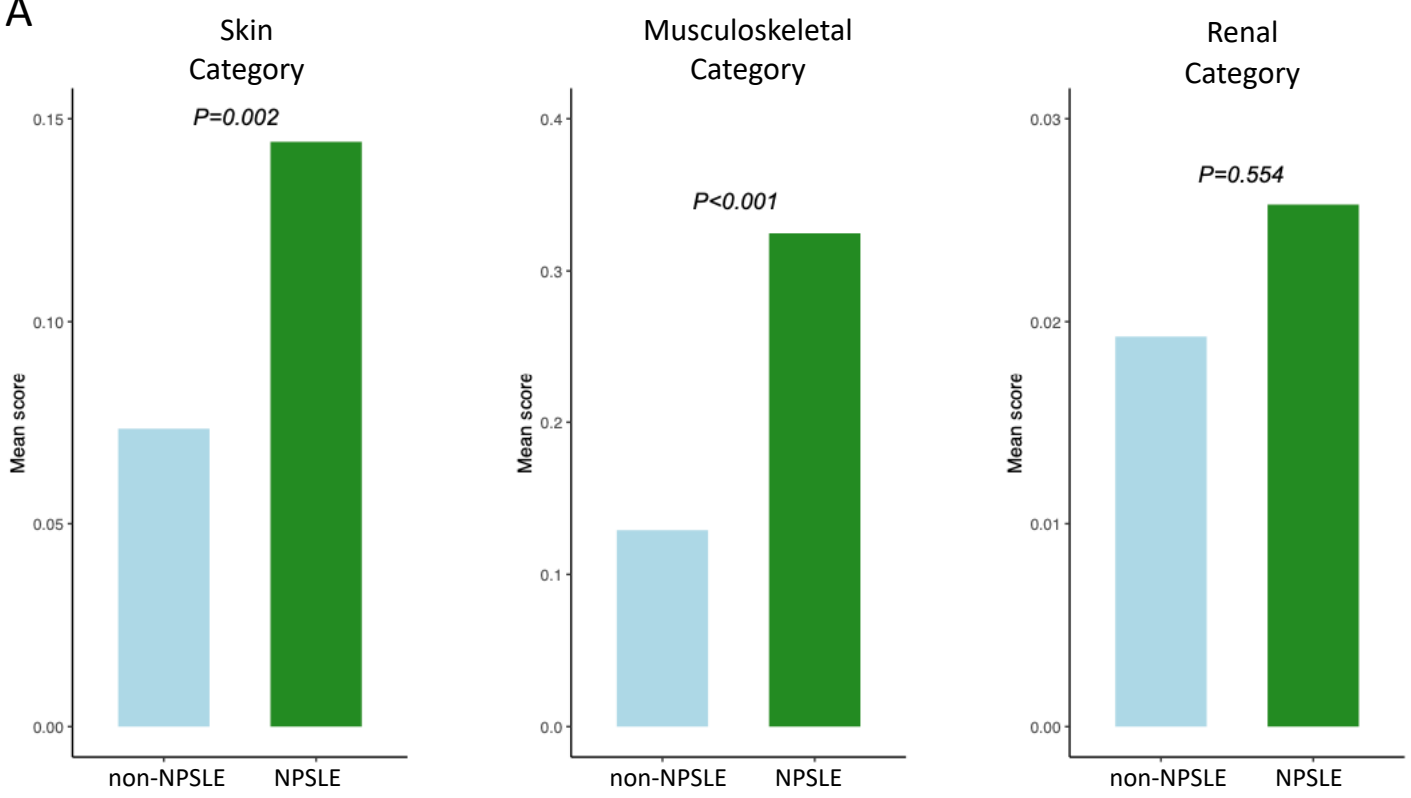

B

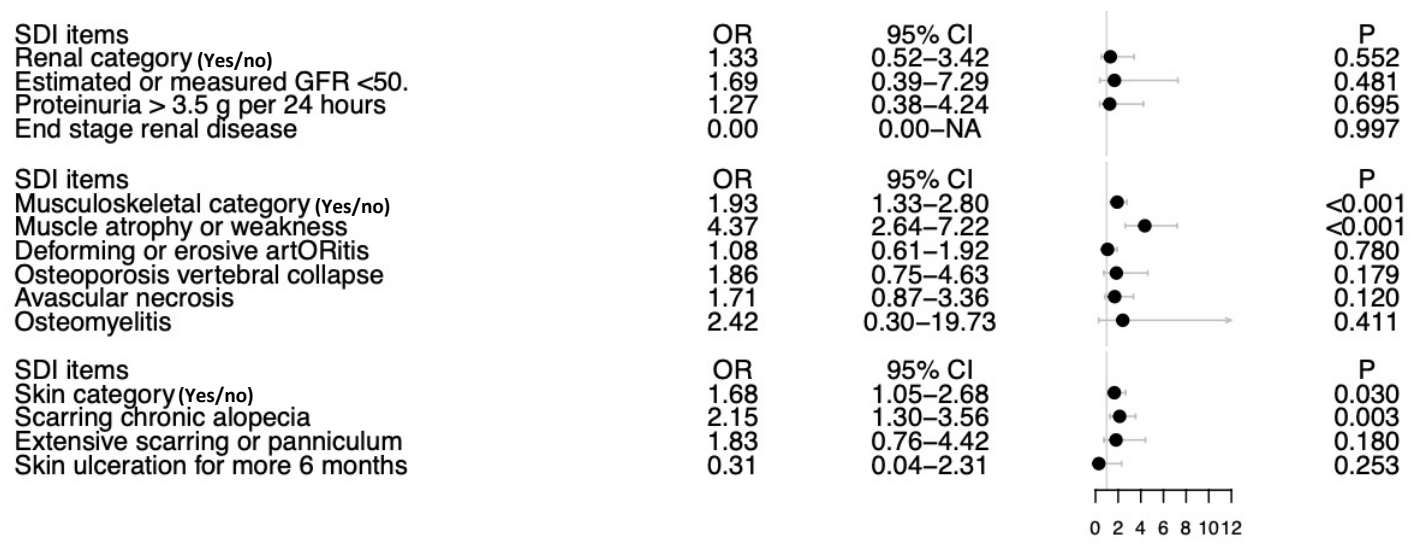

Supplement: Supplementary file 2 — Supplementary file2 (PDF 196 KB) [file 296_2024_5667_MOESM2_ESM.pdf]
