# Supplementary material for: Neuropsychiatric involvement in systemic lupus erythematosus contributes to organ damage beyond the nervous system: a post-hoc analysis of 5 phase III randomized clinical trials
Source: Rheumatol Int. 2024 Aug 8;44(9):1679–89. doi: 10.1007/s00296-024-05667-5 (PMC11343782; doi:10.1007/s00296-024-05667-5)
Supplement: Supplementary file 3 — Supplementary file3 (PPTX 246 KB) [file 296_2024_5667_MOESM3_ESM.pptx]

## Slide 1
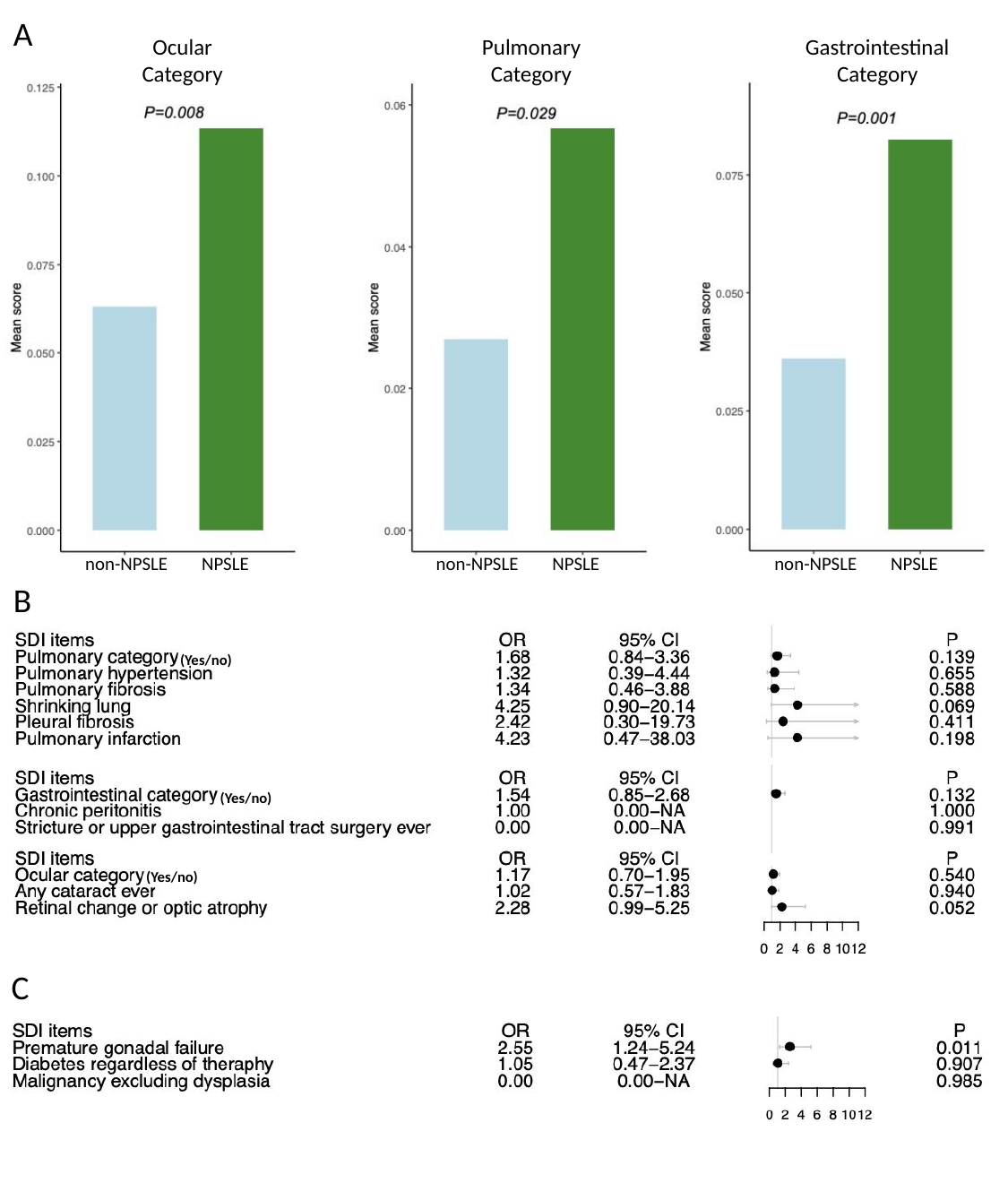

A
Ocular
Category
Pulmonary
Category
Gastrointestinal
Category
non-NPSLE NPSLE
non-NPSLE NPSLE
non-NPSLE NPSLE
B
(Yes/no)
(Yes/no)
(Yes/no)
C
